# Diabetes in relation to biliary tract cancer and stones: a population-based study in Shanghai, China

**DOI:** 10.1038/sj.bjc.6605706

**Published:** 2010-06-01

**Authors:** F M Shebl, G Andreotti, A Rashid, Y-T Gao, K Yu, M-C Shen, B-S Wang, Q Li, T-Q Han, B-H Zhang, J F Fraumeni, A W Hsing

**Affiliations:** 1 Division of Cancer Epidemiology and Genetics, National Cancer Institute, National Institute of Health, Bethesda, MD, USA; 2 Department of Pathology, MD Anderson Cancer Center, Houston, TX, USA; 3 Department of Epidemiology, Shanghai Cancer Institute, Shanghai, China; 4 Shanghai Tumor Hospital, Shanghai, Fudan University, Shanghai, China; 5 Zhongshan Hospital, Shanghai, Fudan University, Shanghai, China; 6 Ruijin Hospital, Shanghai Jiaotong University School of Medicine, Shanghai, China; 7 Institute of Oriental Hepatobiliary Surgery, Second Military Medical University, Shanghai, China

**Keywords:** diabetes, biliary tract cancers, biliary stones, mediation modelling

## Abstract

**Background:**

Biliary tract cancers are rare but fatal malignancies. Diabetes has been related to biliary stones, but its association with biliary tract cancers is less conclusive.

**Methods:**

In a population-based case–control study of 627 cancers, 1037 stones, and 959 controls in Shanghai, China, we examined the association between diabetes and the risks of biliary tract cancer and stones, as well as the effect of potential mediating factors, including serum lipids and biliary stones (for cancer), contributing to the causal pathway from diabetes to biliary diseases.

**Results:**

Independent of body mass index (BMI), diabetes was significantly associated with gallbladder cancer and biliary stones ((odds ratio (OR) (95% confidence interval)=2.6 (1.5–4.7) and 2.0 (1.2–3.3), respectively). Biliary stones and low serum levels of high-density lipoprotein (HDL) were significant mediators of the diabetes effect on gallbladder cancer risk, accounting for 60 and 17% of the diabetes effect, respectively. High-density lipoprotein was also a significant mediator of the diabetes effect on biliary stones, accounting for 18% of the diabetes effect.

**Conclusions:**

Independent of BMI, diabetes is a risk factor for gallbladder cancer, but its effect is mediated in part by biliary stones and serum HDL levels, suggesting that gallbladder cancer risk may be reduced by controlling diabetes, stones, and HDL levels.

Biliary tract cancers, including cancers of the gallbladder, extrahepatic bile duct, and ampulla of Vater, are rare but fatal malignancies (Hsing *et al*, 2006). Other than biliary stones, little is known about the aetiology of biliary tract cancers, although obesity, hyperlipidemia, and diabetes have been suggested as risk factors (Hsing *et al*, 2007, 2008b; Andreotti *et al*, 2008).

Diabetes has been associated with a higher risk of biliary stones in some, but not all, studies (Pagliarulo *et al*, 2004; Festi *et al*, 2008). Diabetes affects serum lipid levels (Abrams *et al*, 1982; Goldberg, 2001), which has a critical role in the development of biliary stones (Carey, 1993; Singh *et al*, 1997). At present, it is unclear whether the association between diabetes and biliary stones is independent of serum lipid levels and whether the diabetes effect on biliary tract cancer is mediated through serum lipid levels and biliary stones.

To clarify these relationships, we examined the association between diabetes and the risks of biliary stones and cancer in Shanghai, China, where the incidence rates of biliary tract cancer have risen sharply in recent decades. We also assessed the potential mediating effects of serum lipid levels and biliary stones on these associations.

## Materials and methods

Details of the study have been reported elsewhere (Hsing *et al*, 2008a, 2008b). Briefly, between 1997 and 2001, patients newly diagnosed with primary biliary tract cancers were recruited through a rapid-reporting system in 42 hospitals in Shanghai. During the study period, the rapid-reporting system identified more than 95% of incident biliary tract cancer patients in Shanghai. Biliary tract cancer cases were confirmed by histopathological assessment (70% of cases) or by medical or surgical records or imaging data (30% of cases). Cancer-free biliary stone patients were identified from the same hospitals as cancer cases, and frequency matched to the index cancer case on age (5-year groups), sex, and diagnosing hospital. Biliary stone cases were confirmed using abdominal ultrasound, endoscopic retrograde cholangiopancreatography, medical or surgical records, or pathologic specimens for those who underwent a cholecystectomy. Healthy subjects without biliary tract cancer were randomly selected from the Shanghai Resident Registry, and were frequency matched to the index cancer case on age (5-year groups) and sex. Biliary stone status for biliary tract cancer cases was determined by self-report (22.7%) or medical records (77.3%), and for population controls it was based on self-report (22.7%) or abdominal ultrasound (77.3%). Participation rates were 95% among cases and 82% among controls. Written informed consents were obtained from all participants, and the study was approved by the institutional review boards of the US National Cancer Institute (NCI) and the Shanghai Cancer Institute (SCI), China.

In-person interviews were conducted by trained interviewers using a structured questionnaire to obtain information on demographic characteristics, lifestyle factors, and medical histories. Cancer and stone cases were interviewed within 3 weeks after diagnosis. The accuracy of the interviews was assessed by randomly selecting 5% of the subjects to be re-interviewed 3 months after the initial interview. Concordance between the two interviews was greater than 90%.

Overnight fasting blood samples were collected from over 80% of the participants who gave consent. Within 4 h of collection, samples were transported to the SCI for processing, and were later shipped to the NCI repository on dry ice by express mail. Fasting serum lipid levels were measured for all subjects who donated overnight fasting blood samples. Blood lipids were measured at the Laboratory of Biochemistry, Institute of Cardiovascular Diseases, Zhongshan Hospital, Shanghai Medical University (Fudan University).

### Statistical analysis

Fisher exact test (when the data were sparse) and *χ*^2^ statistics were used for the bivariate comparisons. Ampulla of Vater and bile duct cancers were compared with all population controls (*n*=959), whereas gallbladder cancer was compared with population controls without cholecystectomy (*n*=902). Biliary stones were compared with population controls without stones (*n*=735). The association between diabetes and biliary tract cancers and stones was assessed using multivariable unconditional logistic regression to calculate the odds ratio (OR) and 95% confidence interval (95% CI) adjusting for potential confounders, including age, sex, body mass index (BMI), waist-to-hip ratio (WHR), education, and aspirin use. Body mass index was grouped according to the WHO classification for Asian populations as normal (18.5–<23 kg m^−2^), overweight (23–<25 kg m^−2^), and obese (⩾25 kg m^−2^) (WHO/IASO/IOTF, 2000; WHO Expert Consultation, 2004). Lipid levels were classified as high triglycerides (⩾1.7 mmol l^−1^), low high-density lipoprotein (HDL) (<1.04 mmol l^−1^), and high low-density lipoprotein (LDL) (⩾4.14 mmol l^−1^) based on the Joint Committee for Developing Chinese Guidelines on Prevention and Treatment of Dyslipidemia in Adults (JCDCG) (2007). Diabetes is based on self-reported history of diabetes.

Mediation modelling was used to assess the percent of total diabetes effect that is mediated by each potential mediating factor. A mediating factor is an intermediate variable in the causal pathway between an independent variable (diabetes) and dependent variable (biliary tract cancer or stones). We examined the potential mediating factors (biliary stones (on cancer risk), total triglycerides, LDL, and HDL (for cancer and stone risk)), which are risk factors for biliary tract cancers/stones and have also been linked to diabetes (Hsing *et al*, 2007; Andreotti *et al*, 2008). Body mass index was not evaluated as a mediator, as obesity probably precedes the development of diabetes and therefore is not in the causal pathway between diabetes and biliary diseases. Instead, BMI was evaluated as a confounder. Structural equation modelling was used to calculate the mediated effect and its statistical significance (MacKinnon *et al*, 2007; Preacher and Hayes, 2008). We ran two mediation analyses: (1) single mediation models (unadjusted, a model that has single mediator), (2) multiple mediation model (adjusted, to obtain the significance of individual mediators within the context of a model that has multiple mediators simultaneously and account for the biological direction between mediators). Triglycerides, LDL, and HDL were used as continuous variables in all mediation modelling. All analyses were conducted using SAS 9.1(SAS Institute, Cary, NC, USA), and Mplus, version 5.

## Results

A total of 368 gallbladder cancer, 191 extrahepatic bile duct cancer, and 68 ampulla of Vater cancer cases, as well as 1037 biliary stone cases and 959 population-based controls, were included in the study. Table 1 shows the characteristics of the study subjects by case–control status.

Prevalence of self-reported diabetes (diagnosed by a doctor in the past) was 8.1% in controls and 13.9, 10.5, and 7.4% in subjects with cancers of the gallbladder, bile duct, and ampulla of Vater, respectively. Diabetes was associated with significant excess risks of gallbladder cancer (OR=2.6, 95% CI 1.5–4.7) and biliary stones (OR=2.0, 95% CI 1.2–3.3), but not with bile duct cancer or ampulla of Vater cancer, after adjustment for age, sex, education, BMI, WHR, duration of diabetes, and aspirin use (Table 2).

**Figure 1:**
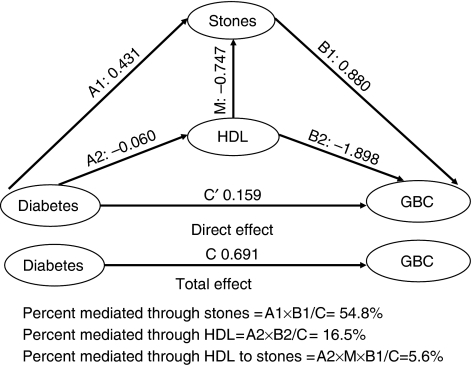
Results of multiple mediation analysis.

In single mediation models, HDL accounted for 17.6% of the effect of diabetes on biliary stone risk (*P*=0.02), and HDL and biliary stones accounted for 22.3% (*P*=0.03) and 44.3% of the diabetes effect on gallbladder cancer risk. When HDL and biliary stones were included in the same model, both factors together explained 76.9% of the diabetes effect on gallbladder cancer risk (*P*<0.0001), with the percent mediated as: (i) diabetes to biliary stones to gallbladder cancer (54.8%), (ii) diabetes to HDL to gallbladder cancer (16.5%), and (iii) diabetes to HDL to biliary stones to gallbladder cancer (5.6%); therefore, 60.4% of the overall diabetes effect on gallbladder cancer risk was mediated through stones (Table 3 and Figure 1).

## Discussion

Data from this population-based case–control study suggest that, independent of BMI, diabetes was associated with excess risks of gallbladder cancer and biliary stones. About 60% of the effect of diabetes on biliary tract cancer was mediated by gallstones and 17% by HDL. High-density lipoprotein is also a significant mediator for gallstones, accounting for 18% of the diabetes effect on gallstone risk.

In our study, independent of BMI, diabetes was associated with a two-fold risk of biliary stones, which is consistent with some previous studies (Chapman *et al*, 1996; Attili *et al*, 1997; De Santis *et al*, 1997; Pagliarulo *et al*, 2004), but not all (Jorgensen, 1989; Loria *et al*, 1994). The inconsistency in these results may be due, in part, to differences in methodology (e.g., patient selection), misclassification of diabetes status (on the basis of self-reported data), or incomplete adjustment for confounding (Feldman and Feldman, 1954; Jorgensen, 1989; Loria *et al*, 1994).

The association between diabetes and biliary stones is biologically plausible, as diabetes has been related to several key factors important in the process of stone formation, including lithogenic bile that is supersaturated with cholesterol, particularly in subjects with dyslipidemia and after initiation of insulin therapy (Abrams *et al*, 1982; Stone and Van Thiel, 1985). Our observation that HDL mediated the effect of diabetes on biliary stones further suggests that diabetes-related dyslipidemia, primarily manifested as lower levels of plasma HDL (Goldberg, 2001), has an important role in predisposing to biliary stones. Low levels of circulating HDL are associated with increased hepatic secretion of cholesterol and diminished secretion of bile salts and phospholipids that contribute to lowering the solubility of cholesterol in bile (Carey, 1993; Volzke *et al*, 2005). Thus, it is possible that the risk of biliary stones may be reduced by increasing circulating levels of HDL through lifestyle modification or medication, especially in patients with diabetes.

Consistent with some previous studies, but not all (Adami *et al*, 1996; Welzel *et al*, 2007; Grainge *et al*, 2009), independent of BMI, we found an excess risk of gallbladder cancer in subjects with diabetes. In the past, it has not been clear whether the diabetes–gallbladder cancer association is independent of gallstones, given the relationship of diabetes to gallstones, and of gallstones to biliary tract cancer. Results of our stratified analysis showed that, irrespective of gallstone status, diabetes is associated with gallbladder cancer, suggesting that diabetes may increase the risk of gallbladder cancer independent of the lipid-stone pathogenesis. Indeed, our mediation analysis indicated that biliary stones explained 60% of the effect of diabetes on gallbladder cancer, whereas HDL accounted for 18%, and only 6% was attributed to HDL via biliary stones pathway. These findings support the role of gallstones in gallbladder cancer aetiology among diabetics. In addition, our findings suggest that HDL may function as a mediator through pathways other than stones, such as inflammation (von Eckardstein *et al*, 2005). Both *in vitro* and *in vivo* studies have shown that low levels of HDL may be related to increased oxidation of LDL, which is associated with increased reactive oxygen species and proinflammatory cytokines (Benitez *et al*, 2006).

Our study has several strengths, including its population-based design, large sample size, high response rate, and thorough evaluation of cancer and stone diagnoses, all of which serve to minimise selection bias and misclassification of outcomes. Limitations of the study should be noted as well. Misclassification of diabetes from self-reported information is likely but should be minimal, as 86% of the subjects who reported having diabetes also reported using antidiabetes treatment. In addition, only 2.2% of the controls who reported no history of diabetes had elevated serum glucose levels, suggesting that few subjects had asymptomatic or undiagnosed diabetes at the time of interview. However, we had no information on cholesterol-lowering drugs, and thus we cannot control for this effect in our analysis of HDL.

In summary, our study revealed an increased risk of gallbladder cancer and biliary stones among diabetics, independent of obesity and other putative risk factors. Both biliary stones and low HDL levels were significant mediators of the diabetes effect on the risk of gallbladder cancer, whereas HDL was a significant mediator of the diabetes effect on biliary stones. These findings need to be confirmed in future studies.

## Figures and Tables

**Table 1:** Selected characteristics of study subjects by case–control status

	**Population controls**	**Biliary tract cancers**			
	**All**	**Gallbladder^a^**	**Extrahepatic bile duct^b^**	**Ampulla of Vater^b^**	**Biliary tract stones^c^**
	***N* (%)**	***N* (%)**	***N* (%)**	***N* (%)**	***N* (%)**
Total	959 (100)	368 (100)	191 (100)	68 (100)	1037 (100)
					
*Age at interview*					
34–49	71 (7.4)	29 (7.9)	18 (9.4)	4 (5.9)	200 (19.3)
50–64	326 (34.0)	116 (31.5)	59 (30.9)	22 (32.4)	405 (39.1)
65–74	562 (58.6)	223 (60.6)	114 (59.6)	42 (61.7)	432 (41.6)
					
*Gender*					
Male	373 (38.9)	99 (26.9)	99 (51.8)	37 (54.4)	390 (37.6)
Female	586 (61.1)	269 (73.1)	92 (48.2)	31 (45.6)	647 (62.4)
					
*Education*					
None/primary	396 (41.3)	198 (53.8)	86 (45.0)	29 (42.7)	317 (30.6)
Middle/high school	423 (44.1)	129 (35.1)	74 (38.7)	31 (45.6)	537 (51.8)
College or higher	140 (14.6)	41 (11.1)	31 (16.2)	8 (11.7)	183 (17.6)
					
*Smoking* d					
No	674 (70.3)	278 (75.8)	115 (60.2)	38 (55.9)	754 (72.7)
Yes	285 (29.7)	89 (24.2)	76 (39.8)	30 (44.1)	283 (27.3)
					
*Alcohol use* e					
No	760 (79.3)	316 (85.9)	141 (73.8)	53 (77.9)	869 (83.9)
Yes	198 (20.7)	52 (14.1)	50 (26.2)	15 (22.1)	167 (16.1)
					
*Body mass index* f					
<18.5	79 (8.3)	17 (4.7)	8 (4.2)	1 (1.47)	44 (4.3)
18.5–22.9	404 (42.2)	130 (35.6)	86 (45.5)	29 (42.7)	350 (33.8)
23.0–24.9	197 (20.5)	73 (20.0)	49 (25.9)	15 (22.1)	259 (25.0)
⩾25.0	278 (29.0)	145 (39.7)	46 (24.3)	23 (33.8)	382 (36.9)
					
*Waist-to-hip ratio* g					
⩽0.81	238 (24.8)	38 (12.2)	21 (13.9)	10 (16.1)	163 (16.0)
0.81–0.852	234 (24.4)	48 (15.4)	24 (15.9)	10 (16.1)	200 (19.7)
0.853–0.897	249 (26.0)	93 (29.8)	40 (26.5)	15 (24.2)	272 (26.8)
>0.897	238 (24.8)	133 (42.6)	66 (43.7)	27 (43.6)	382 (37.6)
					
*Selected medical history*					
Diabetes mellitus					
No	881 (91.9)	316 (86.1)	171 (89.5)	63 (92.7)	925 (89.3)
Yes	78 (8.1)	51 (13.9)	20 (10.5)	5 (7.3)	111 (10.7)
Hypertension					
No	553 (57.7)	230 (62.5)	130 (68.1)	48 (70.6)	695 (67.0)
Yes	406 (42.3)	138 (37.5)	61 (31.9)	20 (29.4)	342 (33.0)
Gallstones					
No	735 (76.6)	60 (16.3)	64 (33.5)	32 (47.1)	0 (0.0)
Yes	224 (23.4)	308 (83.7)	127 (66.5)	36 (52.9)	1037 (100.0)
Triglycerides^h^ (mmol l^−1^)					
<1.7	635 (74.0)	150 (56.8)	52 (36.9)	30 (54.6)	637 (64.9)
⩾1.7	223 (26.0)	114 (43.2)	89 (63.1)	25 (45)	344 (35.1)
HDL^h^ (mmol l^−1^)					
<1.04	292 (34.0)	206 (78.0)	114 (81.4)	46 (83.6)	603 (61.5)
⩾1.04	566 (66.0)	58 (23.0)	26 (18.6)	9 (16.4)	378 (38.5)
LDL^h^ (mmol l^−1^)					
<4.14	776 (90.6)	231 (87.5)	117 (83.0)	44 (80.0)	904 (92.7)
⩾4.14	81 (9.4)	33 (12.5)	24 (17.0)	11 (20.0)	71 (7.3)

Abbreviations: HDL, high-density lipoprotein; LDL, low-density lipoprotein.

a Gallbladder cancer cases were compared with population controls without history of previous cholecystectomy (*n*=902).

b Ampulla of Vater and extrahepatic bile duct cancer cases were compared with all population control (*n*=959).

c Biliary stone cases were compared with controls without biliary stones (*n*=735).

d Ever smoked at least one cigarette per day for 6 months or longer.

e Ever drank alcoholic beverages regularly.

f BMI=weight (kg)/height (m)^2^ 5 years before interview. Categories on the basis of WHO classification for Asians.

g Measured at interview, quartile cutoff points were based on distribution among population controls without history of previous cholecystectomy.

h Cutoff on the basis of the Joint Committee for Developing Chinese Guidelines on Prevention and Treatment of Dyslipidemia in Adults.

**Table 2:** Odds ratios (ORs) and 95% confidence intervals (CIs) for biliary tract cancer and biliary stones in relation to diabetes mellitus^a^

	**Biliary tract cancers**											
	**Gallbladder^b^**			**Extrahepatic bile Duct^c^**			**Ampulla of Vater^c^**			**Biliary tract stones^d^**		
**Diabetes mellitus** a	**Control, *N***	**Cancer, *N***	**OR (95% CI)^e^**	**Control, *N***	**Cancer, *N***	**OR (95% CI)^e^**	**Control, *N***	**Cancer, *N***	**OR (95% CI)^e^**	**Control, *N***	**Cancer, *N***	**OR (95% CI)^e^**
No	834	316	1.0	881	171	1.0	881	63	1.0	688	925	1.0
Yes	68	51	2.63 (1.47–4.68)	78	20	0.79 (0.30–2.07)	78	5	0.74 (0.18–3.07)	47	111	2.03 (1.24–3.33)

a Self-reported diabetes.

b Gallbladder cancer cases were compared with population controls without history of previous cholecystectomy.

c Extrahepatic bile duct and ampulla of Vater cancer cases compared with all population controls.

d Bile duct and gallbladder stone cases compared with controls without biliary stones.

e Adjusted for age, sex, education, diabetes duration, body mass index, waist-to-hip ratio, and aspirin use.

**Table 3:** Mediation of the effect of diabetes mellitus^a^ on biliary tract cancers and stones through HDL, LDL, total triglycerides, and gallstones

	**Biliary stones**		**Gallbladder cancer**	
	**Percent mediated^b^**	***P*-value^c^**	**Percent mediated^b^**	***P*-value^c^**
*Mediators in single mediation models* d				
HDL	17.6%	0.02	22.3%	0.03
LDL	NA^e^	0.51	NA^e^	0.57
Total triglycerides	NA^e^	0.09	NA^e^	0.10
Biliary stones	NA^f^	NA^f^	44.3%	<0.0001
				
*Mediators in multiple mediation model* g				
HDL			16.5%	0.03
HDL to biliary stones			5.6%	<0.0001
Biliary stones			54.8%	0.02
Total mediated effects^h^			76.9%	<0.0001

Abbreviations: HDL, high-density lipoprotein; LDL, low-density lipoprotein.

a Self-reported diabetes.

b Percent of total diabetes effect that is mediated.

c Calculated using 5000 bootstrap samples.

d Calculated using structural equation modelling.

e Not applicable because the pathway is not significant.

f Not applicable because biliary stones is the outcome.

g Calculated using structural equation modelling. HDL adjusted for biliary stones, and vice versa.

h Sum of percent mediated by different mediators.
